# Chromosome level genome assembly of colored calla lily (*Zantedeschia elliottiana*)

**DOI:** 10.1038/s41597-023-02516-1

**Published:** 2023-09-09

**Authors:** Yi Wang, Tuo Yang, Di Wang, Rongxin Gou, Yin Jiang, Guojun Zhang, Yuhong Zheng, Dan Gao, Liyang Chen, Xiuhai Zhang, Zunzheng Wei

**Affiliations:** 1 Institute of Grassland, Flowers and Ecology, Beijing Academy of Agriculture and Forestry Sciences, Beijing, 100097 China; 2 College of Horticulture, China Agricultural University, Beijing, 100193 China; 3 College of Horticultural Science & Technology, Hebei Key Laboratory of Horticultural Germplasm Excavation and Innovative Utilization/Hebei Higher Institute Application Technology Research and Development Center of Horticultural Plant Biological Breeding, Hebei Normal University of Science & Technology, Qinhuangdao, 66004 China; 4 Institute of Botany, Jiangsu Province and Chinese Academy of Sciences, Nanjing Botanical Garden, Mem. Sun Yat-Sen, Nanjing, 210014 China; 5 Smartgenomics Technology Institute, Tianjin, 301700 China

**Keywords:** Plant evolution, Plant genetics

## Abstract

The colored calla lily is an ornamental floral plant native to southern Africa, belonging to the Zantedeschia genus of the Araceae family. We generated a high-quality chromosome-level genome of the colored calla lily, with a size of 1,154 Mb and a contig N50 of 42 Mb. We anchored 98.5% of the contigs (1,137 Mb) into 16 pseudo-chromosomes, and identified 60.18% of the sequences (694 Mb) as repetitive sequences. Functional annotations were assigned to 95.1% of the predicted protein-coding genes (36,165). Additionally, we annotated 469 miRNAs, 1,652 tRNAs, 10,033 rRNAs, and 1,677 snRNAs. Furthermore, Gypsy-type LTR retrotransposons insertions in the genome are the primary factor causing significant genome size variation in Araceae species. This high-quality genome assembly provides valuable resources for understanding genome size differences within the Araceae family and advancing genomic research on colored calla lily.

## Background & Summary

*Zantedeschia* spp, commonly known as calla lily, is a perennial herbaceous flowering plant belonging to genus Zantedeschia of the family Araceae. It is typically found in swamps and hills regions of South Africa^1,2^. Through its unique spathes and decorative foliage, calla lily has become popular tubers flowering plants worldwide. It is usually divided into two groups: white calla lily and colored calla lily^3^. Colored calla lily is a significant economic horticultural crop that have been among the top cut flower and tuber exports in New Zealand for the past three decades, while also contributing substantially to the horticultural export revenues of the Netherlands and the United States. Furthermore, the tubers of colored calla lilies have medicinal value and are effective in treating certain gastrointestinal and trauma-related illnesses.

**Fig. 1:**
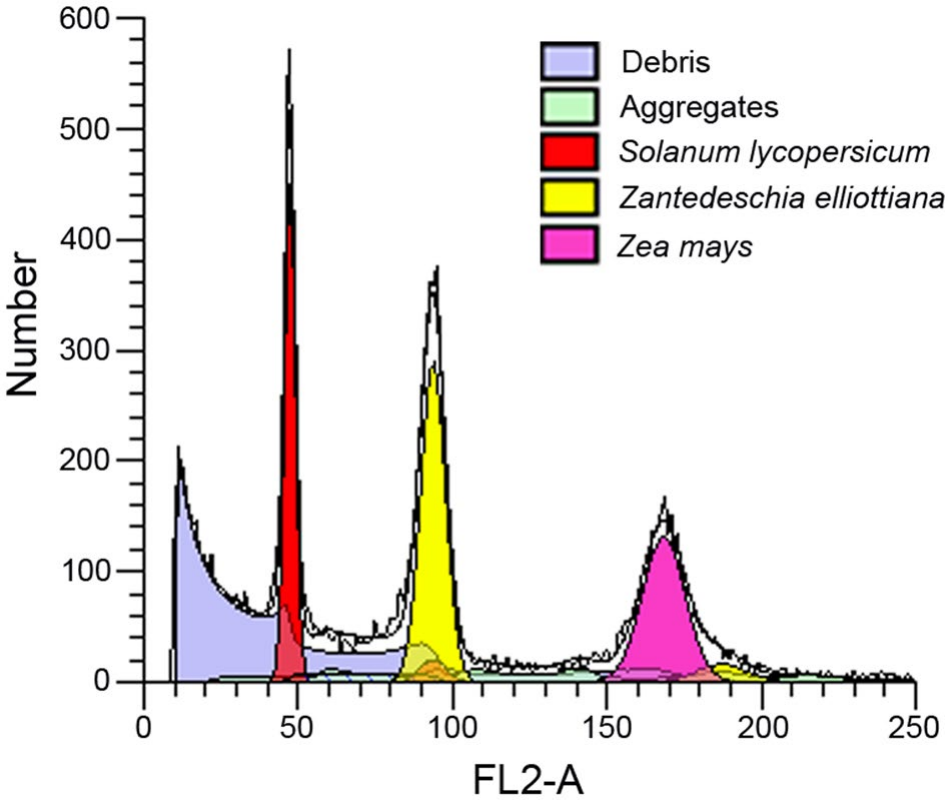
Genome size estimation of *Zantedeschia elliottiana* cv. ‘Jingcai Yangguang’ by flow cytometry. Tomato and maize were used as internal references to genome size estimation.

**Fig. 2:**
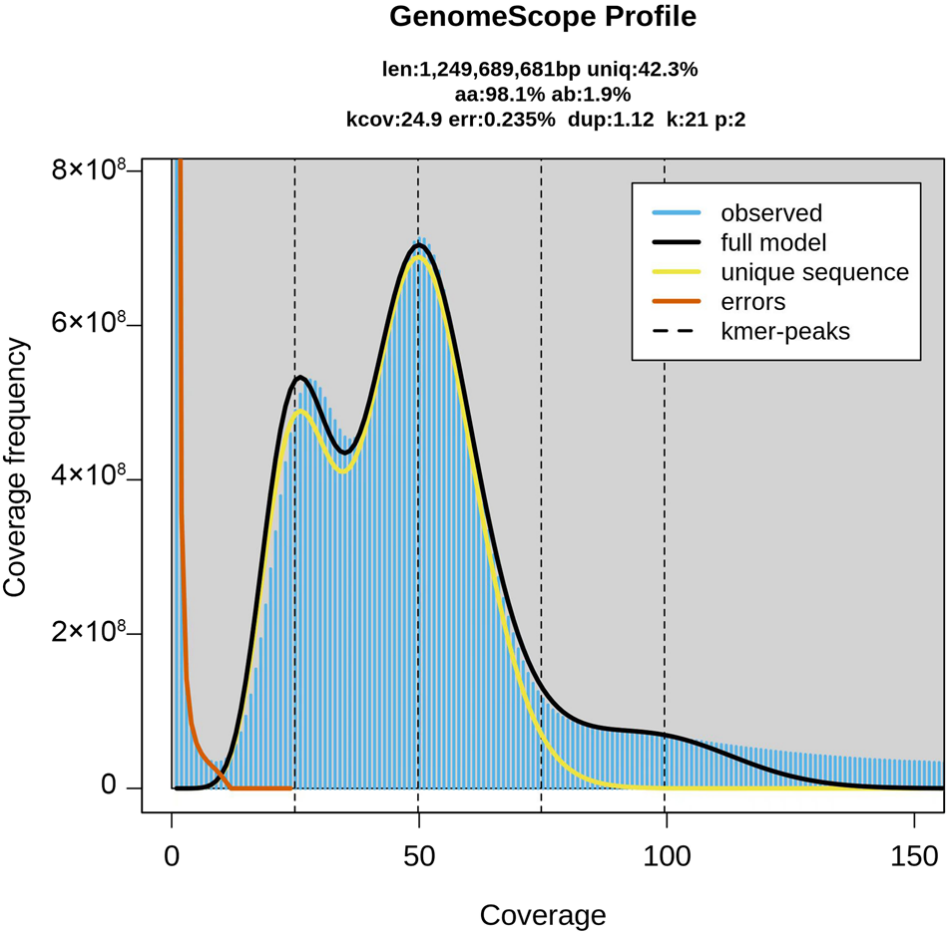
Genome size estimation of *Zantedeschia elliottiana* cv. ‘Jingcai Yangguang’ using Illumina reads.

**Fig. 3:**
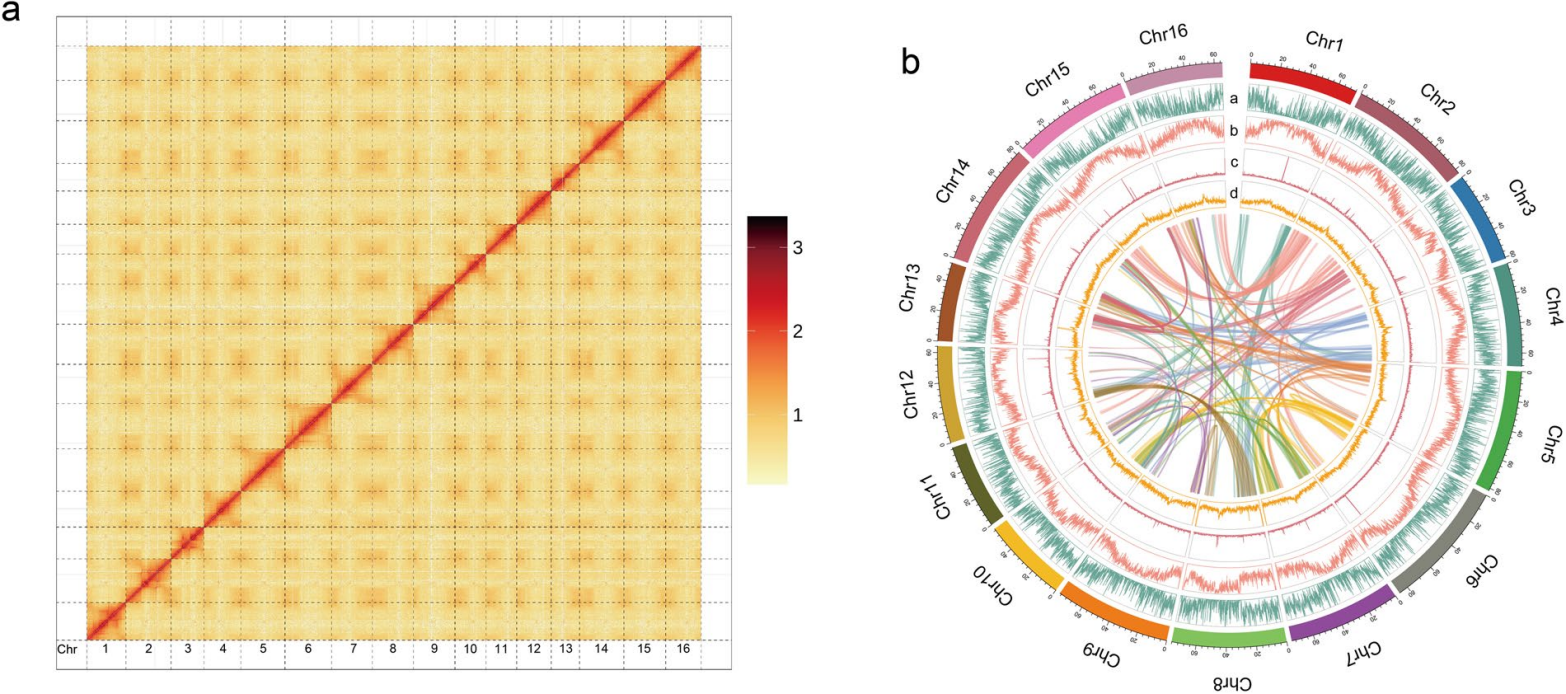
Characteristics of *Zantedeschia elliottiana* cv. ‘Jingcai Yangguang’ genome. (**a**) Hi-C heatmap of the *Zantedeschia elliottiana* cv. ‘Jingcai Yangguang’ genome. (**b**) Circos plot of *Zantedeschia elliottiana* cv. ‘Jingcai Yangguang’ genome. (**a**) Gene density, (**b**) TE density, (**c**) Tandem repeats density, (**d**) GC content and syntenic blocks.

**Fig. 4:**
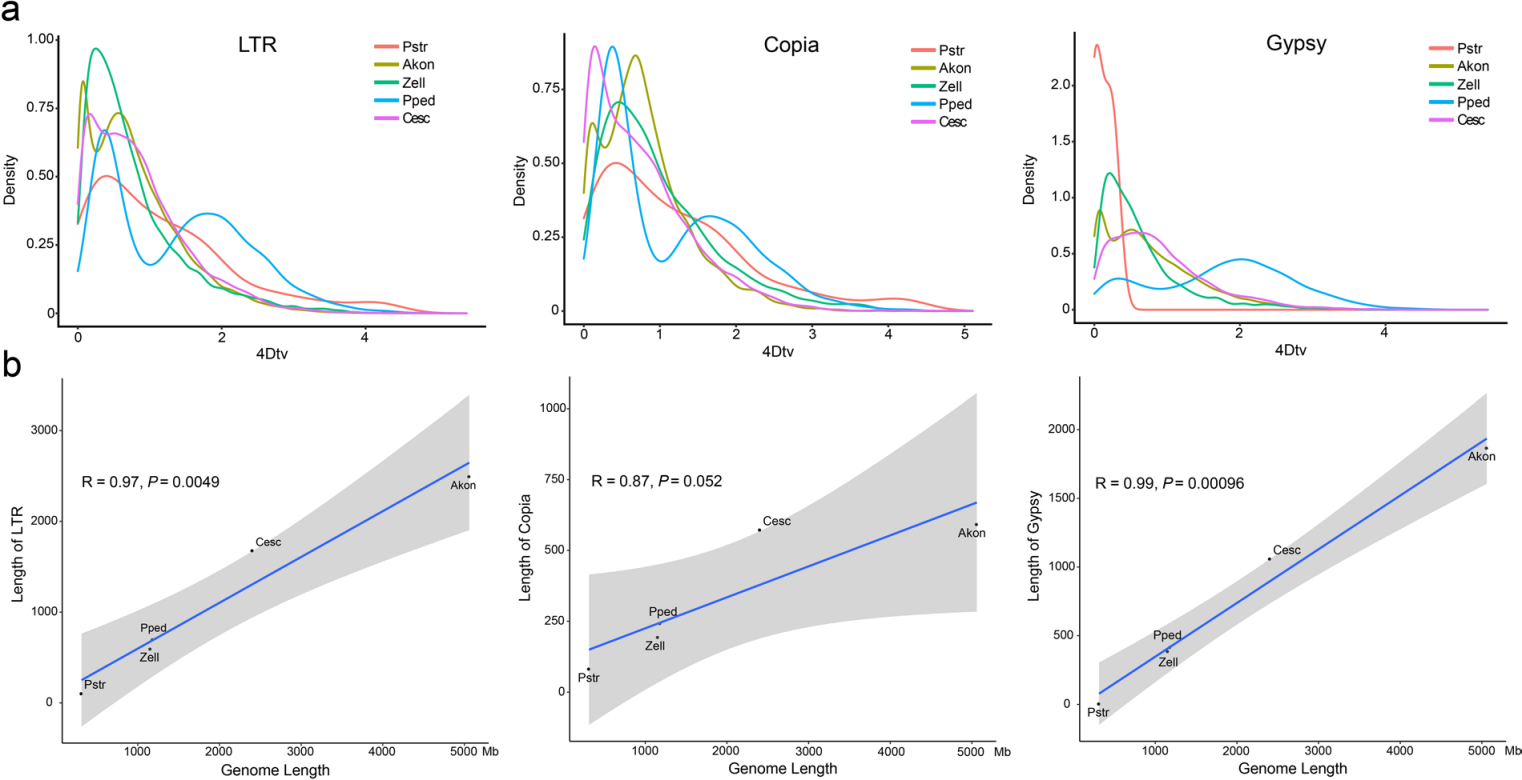
The influence of LTRs on genome size. (**a**) The insertion time of LTRs (Copia and Gypsy) was predicted by 4Dtv. Pstr, *Pistia stratiotes*; Akon, *Amorphophallus konjac*; Zell, *Zantedeschia elliottiana* cv. ‘Jingcai Yangguang’; Pped, *Pinellia pedatisecta*; Cesc, *Colocasia esculenta*. (**b**) Analysis of the correlation between the total length of LTRs and the genome size.

Through k-mer and flow cytometry analysis, the genome size of *Zantedeschia elliottiana* cv. ‘Jingcai Yangguang’ was ~1.2 Gb, with a genome heterozygosity of 1.9% and a repeat sequence proportion of 67.84% (Figs. 1, 2). The *de-novo* assembly of the genome used 84.30X Illumina paired-end short reads (100.31 Gb), 36.92X HiFi reads (43.93 Gb) and 141.45X Hi-C reads (168.18 Gb). We first assembled the genome by HiFi reads and generated a 1,154 Mb contig sequence with 42 Mb contig N50 size (Table 1). Using Hi-C reads, 98.50% of the contigs were anchored into 16 pseudo-chromosomes (Fig. 3, Table 1). The transposable elements content of the total genome in the final annotation is 60.18%, of which LTR retroelement accounted for the largest proportion (51.54%). On the contrary, the proportion of DNA transposons was only 3.73% (Table 2). A total of 36,165 protein-coding genes were predicted, of which 95.1% could be functionally annotated through the InterPro^4^, Pfam^5^, Swiss-Prot^6^, NCBI Non-redundant protein (NR)^7^ and Kyoto Encyclopedia of Genes and Genomes (KEGG)^8^ databases (Table 3). In addition, 10,033 rRNA, 1,677 snRNA, 469 miRNA and 1,652 tRNA in *Zantedeschia elliottiana* cv. ‘Jingcai Yangguang’ genome were obtained by non-coding RNA annotation (Table 4). Using BUSCO evaluation, 98% of the core genes can be identified, including 95.7% of complete single-copy genes and 2.3% of duplicated genes (Table 1). 93.83~95.23% of RNA-seq reads from eight *Zantedeschia elliottiana* cv. ‘Jingcai Yangguang’ tissues (tuber, leaf, pistil, root, spathe, stamen, stem and style) could be mapped to the genome. 99.02% of Illumina reads and 98.42% of HiFi reads were correctly mapped to the genome. The LTR Assembly Index (LAI) of the genome was 18.43, which directly proved that the genome has high continuity (Table 1). LTR insertion time analysis showed that Araceae plants had different LTR bursts during genome evolution, and different types of LTR have different burst states. For Copia-type LTR retrotransposons, *Pistia stratiotes* and *Zantedeschia elliottiana* cv. ‘Jingcai Yangguang’ had the same insertion time. Interestingly, *Amorphophallus konjac* and *Colocasia esculenta* experienced two outbreaks of Copia and Gypsy. The time interval between the two outbreaks of *Colocasia esculenta* were obvious, while *Amorphophallus konjac* were close. Analysis also showed that Gypsy of *Pistiastratiotes* had recently experienced an outbreak (Fig. 4a). As a branch of Araceae family, Lemnaceae plantshave a smaller genome size and number of genes than True-Araceae plants. However, the genome size of True-Araceae plants is not related to the number of genes. Correlation analysis further explained the high correlation between genome size and transposable elements. Gypsy-type LTR retrotransposons had the highest correlation with genome size (Fig. 4b).

**Table 1:** Summary of the *Z. elliottiana* genome.

Assembly characteristics	*Z. elliottiana* cv. ‘Jingcai Yangguang’
**Total length of contigs (bp)**	1,154,500,755
**N50 length of contigs (bp)**	42,376,536
**Total number of contigs**	375
**Longest contigs**	80,375,493
**Total gap size (bp)**	6,700
**Total sequences anchored to the pseudo-chromosomes (bp)**	1,137,238,020
**place rate (%)**	98.50
**Number of annotated genes**	36,165
**Percentage of transposon element sequences (%)**	60.18
**Complete BUSCOs (%)**	98.00
**Fragmented BUSCOs (%)**	0.80
**Missed BUSCOs (%)**	1.20
**LAI**	18.43

**Table 2:** Classification of repetitive sequences in *Z. elliottiana* cv. ‘Jingcai Yangguang’ genome.

*Z. elliottiana* cv. ‘Jingcai Yangguang’		
Without N gaps: 1,154,500,755		
Repetitive sequences	Length (bp)	Ratio (%) in genome
**LTR Retroelement**	595,082,160	51.54
**Gypsy (LTR)**	387,224,277	33.54
**Copia (LTR)**	194,442,787	16.84
**LINE**	47,578,872	4.12
**SINE**	293,029	0.03
**DNA transposons**	43,021,358	3.73
**Other/Unspecified/Unknown**	29,613,899	2.57

**Table 3:** Statistics of gene functional annotation.

Database	*Z. elliottiana* cv. ‘Jingcai Yangguang’	
	Gene numbers	Ratio (%)
**NR**	23,081	63.80
**Swiss-Prot**	16,854	46.60
**KEGG**	16,690	46.10
**Pfam**	19,198	53.10
**GO**	15,276	42.20
**Annotated**	34,406	95.10
**Total**	36,165	—

**Table 4:** Classification of non-coding RNAs in *Z. elliottiana* cv. ‘Jingcai Yangguang’ genome.

Type		Number	Average length (bp)	Total length (bp)	% of genome
**miRNA**		469	117.95	55,320	0.004792
**tRNA**		1,652	75.21	124,254	0.010763
**rRNA**	**18S**	1,571	1759.02	2,763,428	0.239360
	**28S**	6,091	143.58	874,541	0.075750
	**5.8S**	1,532	159.30	244,048	0.021139
	**5S**	839	115.46	96,874	0.008391
**snRNA**	**CD-box**	1,408	106.49	149,933	0.012987
	**HACA-box**	69	147.61	10,185	0.000882
	**splicing**	200	134.86	26,971	0.002336

Here, a high-quality chromosome-level assembly of *Zantedeschia elliottiana* cv. ‘Jingcai Yangguang’ was assembled, revealing the fundamental cause of genome size variation in the Araceae family.

## Methods

### Sample collection and sequencing

‘Jingcai Yangguang’ is a variant of *Zantedeschia elliottiana* cv. ‘Black Magic’ with a chromosome number of 2n = 2x = 32. It was initially cultivated in 2015 by Di Zhou, a former associate researcher in our team. Its young leaves were collected for genome sequencing, and the sequencing material was sourced from the same plant to ensure accuracy of the sequencing. Eight tissues (tuber, leaf, pistil, root, spathe, stamen, stem and style) were sampled for transcriptome sequencing, and the sequencing results were used for gene structure annotation.

The FastPure Plant DNA Isolation Mini Kit (Vazyme, CHN) was employed for DNA extraction from leaf tissue. In liquid nitrogen, fresh leaves were pulverized into a fine powder, and genomic DNA was isolated according to the manufacturer’s guidelines. NanoDrop 2000 (Thermo Scientific, USA) and gel electrophoresis were utilized to evaluate the concentration and purity of the isolated DNA.

The high-quality DNA was used to construct a genomic library, and the library construction and sequencing work were completed at Novogene Co., Ltd. in Beijing. The library is then size-selected using BluePippin (Sage Science, USA) to obtain fragments of the desired size range, which is typically ~15 kb for HiFi sequencing. The purified and size-selected library is then sequenced on the PacBio Sequel II system (Pacifc Biosciences, USA). For Illumina sequencing, a short-read sequencing library was constructed with an insert size of ~250 bp and sequenced on an Illumina NovaSeq. 6,000 platform (Illumina, USA). The Hi-C library was constructed using the same leaf sample as previously described. Briefly, nuclear DNA was fixed with formaldehyde and digested with the restriction enzyme DpnII (NEB, UK). Biotinylated nucleotides were added to the termini of the fragmented DNA, followed by enrichment and size selection to obtain fragments approximately 500 bp. The library was sequenced on the Illumina NovaSeq. 6,000 platform (Illumina, USA).

The RNAprep Pure Plant Kit (TIANGEN, CHN) was used to extract RNA from 8 different tissues (tuber, leaf, pistil, root, spathe, stamen, stem and style). The tissue samples were ground with liquid nitrogen and lysis buffer was added to extract RNA. The RNA was isolated according to the manufacturer’s guidelines. RNA-seq libraries were generated and sequenced on an NovaSeq. 6,000 platform (Illumina, USA).

### Genome size estimation

Two methods, k-mer and flow cytometry analysis, were employed to estimate the genome size of *Zantedeschia elliottiana* cv. ‘Jingcai Yangguang’. For flow cytometry analysis, the DNA content of *Zantedeschia elliottiana* cv. ‘Jingcai Yangguang’ was assessed using the BD Accuri C6 flow cytometer (BD Biosciences, USA), with tomato and maize as reference standards (Fig. 1). The frequency distribution of k-mer was assessed using Jellyfish (v1.0.0) (-C -m 21 -G 2)^9^. Using GenomeScope (v2.0) (-p 2 -k 21)^10^ to calculate the genome size and heterozygosity level with k-mer size = 21 (Fig. 2).

### *De-novo* genome assembly

Firstly, contigs were assembled from HiFi reads using hifiasm (v0.19.5) (https://github.com/chhylp123/hifiasm) with default parameters. Subsequently, Hi-C reads were aligned to contigs using HICUP (v0.7.3)^11^ to evaluate the efficiency of data. Following that, contigs were anchored into 16 pseudo-chromosomes using YaHS (v1.1) with default parameters (Fig. 3). Finally, the assembled genome was manually corrected with Juicebox (v1.11.08) (Table 1)^12^.

### Completeness evaluation of the assembled genome

Benchmarking Universal Single-Copy Orthologs (BUSCO v5.4.5, embryophyta_odb10)^13^, and LTR Assembly Index (LAI, LTR_retriever v2.9.0)^14^ were used to determine the completeness of the genome, respectively (Table 1).

### Genome prediction and annotation

The annotation pipeline employed for predicting repeat elements consisted of both homology-based and *de-novo* approaches. In the homology-based approach, alignment searches were conducted against the Repbase database (http://www.girinst.org/repbase)^15^ to identify homologous evidence, which was subsequently predicted using RepeatProteinMask (v4.1.0) (http://www.repeatmasker.org/). For *de-novo* annotation, a *de-novo* library was constructed using LTR_FINDER (v1.07)^16^, RepeatScout (v1.0.6) (http://www.repeatmasker.org/)^17^, and RepeatModeler (v2.0.4) (http://www.repeatmasker.org/RepeatModeler.html)^18^. The annotation process was then performed using Repeatmasker (v4.1.0) (http://repeatmasker.org/)^19^.

To annotate the gene structure, a strategy incorporating *de-novo* prediction, protein-based homology, and transcriptome were employed. Protein sequences from *Amorphophallus konjac*, *Colocasia esculenta*, *Lemna minuta*, *Spirodela polyrhiza*, *Pistia stratiotes* and *Pinellia pedatisecta* were mapped to their respective genome using WUblast (v2.0)^20^. GeneWise (v2.4.1)^21^ was utilized to predict the gene structures in the genomic regions identified by WUblast (v2.0). The gene structures generated by GeneWise (v2.4.1) were referred to as the Homo-set. Additionally, gene models produced by PASA (v2.5.2)^22^, which served as training data for *de-novo* gene prediction programs. Five *de-novo* gene prediction programs, namely AUGUSTUS (v2.5.5)^23^, Genscan (v1.0)^24^, Geneid (v1.4)^25^, GlimmerHMM (v3.0.1)^26^ and SNAP (v2013.11.29)^27^, were employed to predict coding regions within the repeat-masked genome. To perform transcript-based annotations, the clean data were aligned to the genome assembly using TopHat (v2.0)^28^, and Cufflinks (v2.1.1)^29^. These results were combined by EVidenceModeler (v1.1.1)^22^, which generated a non-redundant set of gene annotations.

The predicted protein sequences were functionally annotated through searches in five databases: NR^7^, InterPro^4^, KEGG^8^, Pfam^5^ and Swiss-Prot^6^. Gene Ontology (GO)^30^ annotation was performed using InterProScan (v5.52–86.0)^31^ (Table 3). Blast (v2.2.26) (E-value threshold of 1E-5) were used to align the protein sequences of *Zantedeschia elliottiana* to these databases for gene function annotation.

Noncoding RNA (ncRNA) annotation was conducted using tRNAScan (v1.4)^32^ and blast (v2.2.26)^33^ for predicting tRNA and rRNA, respectively. Furthermore, miRNA and snRNA were identified through alignment with the Rfam database^34^ using INFERNAL (v1.0)^35^.

### Estimation of LTR retrotransposons insertion timing

The full-length LTR retrotransposons were aligned to the ClariTeRep^36^ datasets using blastn (blast, v2.2.26). The insertion time of each LTR retrotransposon was calculated. The alignment of the 5’ and 3’ LTRs was performed using MUSCLE (v5.1)^37^, and the EMBOSS software package (v6.6.0)^38^ was used to calculate the accumulated divergence^39^.

## Data Records

The raw data (PacBio HiFi reads, Illumina reads, and Hi-C sequencing reads) used for genome assembly were deposited in the SRA at NCBI SRR24273711-SRR24273714^40–43^.

The RNA-seq data were deposited in the SRA at NCBI SRR24273483-SRR24273490^44–51^. The genome assembly and annotation files are available in Figshare (10.6084/m9.figshare.22656112)^52^ and GenBank under the accession JARZZO000000000^53^.

## Technical Validation

Firstly, the Hi-C heatmap exhibits the accuracy of genome assembly, with relatively independent Hi-C signals observed between the 16 pseudo-chromosomes (Fig. 2a). Moreover, we aligned RNA and DNA reads to the final determined genome to assess the accuracy of genome assembly. For the alignment of DNA reads, Illumina reads were aligned using BWA (v0.7.17)^54^ with default parameters, while HiFi reads were aligned using minimap2 (v2.24-r1122)^55^ with default parameters. The mapping rate for Illumina reads was 99.02%, while the mapping rate for HiFi reads was 98.42%. For the alignment of RNA reads, transcriptomic data from different tissues were individually mapped to the final determined genome using HISAT2 (v2.2.1)^56^ with default parameters. The mapping rates for the respective tissue-specific transcriptomic data ranged from 93.83% to 95.23%. Furthermore, we evaluated the completeness of the genome using BUSCO (v5.4.5, embryophyta_odb10)^13^, and LAI (LTR_retriever, v2.9.0)^14^ (Table 1). Overall, these assessments individually confirmed the accuracy and completeness of the genome assembly.

## Data Availability

All data processing commands and pipelines were carried out in accordance with the instructions and guidelines provided by the relevant bioinformatic software. There were no custom scripts or code utilized in this study.
